# Hypothermia and nutrition: at present more questions than answers?

**DOI:** 10.1186/cc11286

**Published:** 2012-06-07

**Authors:** Ronny Beer, Marlene Fischer, Anelia Dietmann, Bettina Pfausler, Erich Schmutzhard

**Affiliations:** 1 Neurological Intensive Care Unit, Department of Neurology, Innsbruck Medical University, Innsbruck, Austria

## 

Therapeutic hypothermia (TH) has been recently accepted as a powerful medical intervention for providing neuroprotection to patients sustaining cardiac arrest and hypoxic-ischemic encephalopathy. Further, TH has also been explored as a potential treatment strategy for patients with traumatic brain injury and ischemic or hemorrhagic stroke (for review see [1]). With the widespread propagation of TH as treatment for intracranial hypertension in patients who are refractory to standard interventions [2], induction and maintenance of TH is no longer restricted to the early period (that is, the first 12 to 24 hours) of acute brain injuries but has expanded over several days until rewarming [3]. Therefore, it is evident that, in addition to the disease process itself, TH has a major impact on ICU management such as analgesia and sedation, ventilator therapy, cardiocirculatory support, or artificial nutrition.

Importantly, the significance of nutrition therapy in critically ill patients cannot be overstated [4]. Critical illness typically induces a catabolic stress state proportional to the severity of injury, predisposing patients to serious nutritional deficits coupled with multiple organ dysfunction, delayed recovery and disproportionate mortality [5]. Effective nutrition therapy can play a major role in attenuating the catabolic response and avoiding the harmful effects of prolonged hypermetabolism. International guidelines provide recommendations for timing until initiation of artificial nutrition, administration route, energy targets and type of nutrients in critically ill patients [4,6]. Starting early nutrition within 24 to 48 hours, primarily using the enteral route, is a proactive strategy to reduce energy deficit [7]. Importantly, underfeeding and overfeeding must be avoided, which implies monitoring nutritional delivery to timely identify an increasing energy gap or excess administration [8]. Energy requirements are most accurately assessed by measuring resting energy expenditure using indirect calorimetry; however, this method is not widely available [9]. Instead, guideline targets could be applied, with a cautious initial energy requirement of 20 to 25 kcal/kg/day increasing thereafter in the recovery phase. Enteral nutrition has the advantage of preventing adverse structural and functional alterations of the gut barrier and of improving mesenteric blood flow as well as enhancing local and systemic immune responsiveness [10]. On the other hand, critically ill patients may often have intolerance to gastric feeding [11]. Prokinetic medications may help restore gastric emptying and promote gastrointestinal motility [12]. Persistent intolerance to enteral nutrition selects patients who will require supplemental parenteral nutrition [6]. Another issue in the context of metabolic consequences of nutrition therapy that deserves attention is glycemic control. Both hyperglycemia and hypoglycemia are of particular concern in critically ill patients [13]. However, to date the goal for blood glucose level and whether intensive or a moderate insulin therapy should be employed in these patients, especially with acute brain injuries, is still uncertain [14]. Implementation of a protocol to promote control of serum glucose when providing nutrition therapy is advocated, and a range of 110 to 150 mg/dl (6.1 to 8.3 mmol/l) may be most appropriate [4].

Whether the aforementioned recommendations on artificial nutrition of the general intensive care patient population can also be applied on equal terms to patients undergoing TH is still a matter of debate. Studies addressing the various issues of nutrition therapy in TH are scarce, as evidenced by a contemporary PubMed search including bibliographies of published reviews. So far, data are available on the course of energy expenditure in patients treated with TH suffering from ischemic or hemorrhagic stroke [15,16] and severe traumatic brain injury [2]. In sedated, ventilated patients receiving muscle relaxants a significant decrease in resting energy expenditure ranging at approximately 75 to 85% of baseline values during TH could be demonstrated. Downregulation of cerebral and overall metabolism as well as muscle relaxation has been discussed as major factors of the reduction in energy expenditure [15]. In contrast, shivering, an anticipated consequence and potentially major adverse effect of TH, has been shown to be strongly associated with graded increases in systemic metabolism [16]. This clearly indicates the need for an individualized determination of the optimal amount of energy to be delivered during TH. Further, one has to bear in mind that other (patho)physiological changes might occur in patients treated with TH. During the maintenance period of TH, electrolyte replacement is often needed because of a decrease in serum levels of potassium, magnesium and phosphate. With rewarming, these electrolytes are released from intracellular stores and move to extracellular spaces. Therefore, care must be taken to avoid rebound hyperkalemia [17]. In addition, insulin resistance, which can lead to hyperglycemia, may occur during TH. Again, in the rewarming period insulin sensitivity may increase rapidly, resulting in sometimes marked hypoglycemia if the insulin dose is not adjusted appropriately [17].

In conclusion, many questions regarding the nutrition therapy of patients treated with TH remain to be answered. Further studies that focus on the optimal amount of caloric intake, timing, preferred route of administration and monitoring of nutrition delivery during targeted temperature management are urgently needed.

## Competing interests

RB received financial support as speaker's fees from Baxter Austria and Fresenius-Kabi Austria.
